# Maternal immune activation evoked by polyinosinic:polycytidylic acid does not evoke microglial cell activation in the embryo

**DOI:** 10.3389/fncel.2015.00301

**Published:** 2015-08-05

**Authors:** Silke Smolders, Sophie M. T. Smolders, Nina Swinnen, Annette Gärtner, Jean-Michel Rigo, Pascal Legendre, Bert Brône

**Affiliations:** ^1^BIOMED – Hasselt UniversityHasselt, Belgium; ^2^Laboratory of Neuronal Differentiation, VIB Center for the Biology of Disease, Leuven and Center for Human Genetics, KU LeuvenLeuven, Belgium; ^3^INSERM, UMR S 1130, Université Pierre et Marie CurieParis, France; ^4^CNRS, UMR 8246, Université Pierre et Marie CurieParis, France; ^5^UM 119 NPS, Université Pierre et Marie CurieParis, France

**Keywords:** neuropsychiatric disorders, maternal immune activation, microglia, embryo, cortex

## Abstract

Several studies have indicated that inflammation during pregnancy increases the risk for the development of neuropsychiatric disorders in the offspring. Morphological brain abnormalities combined with deviations in the inflammatory status of the brain can be observed in patients of both autism and schizophrenia. It was shown that acute infection can induce changes in maternal cytokine levels which in turn are suggested to affect fetal brain development and increase the risk on the development of neuropsychiatric disorders in the offspring. Animal models of maternal immune activation reproduce the etiology of neurodevelopmental disorders such as schizophrenia and autism. In this study the poly (I:C) model was used to mimic viral immune activation in pregnant mice in order to assess the activation status of fetal microglia in these developmental disorders. Because microglia are the resident immune cells of the brain they were expected to be activated due to the inflammatory stimulus. Microglial cell density and activation level in the fetal cortex and hippocampus were determined. Despite the presence of a systemic inflammation in the pregnant mice, there was no significant difference in fetal microglial cell density or immunohistochemically determined activation level between the control and inflammation group. These data indicate that activation of the fetal microglial cells is not likely to be responsible for the inflammation induced deficits in the offspring in this model.

## Introduction

Schizophrenia and autism are neurodevelopmental disorders that can arise early during postnatal life. Although genetic deficits are important risk factors, perturbations of local environment, especially during pregnancy, are suspected to play a central role in the occurrence of these neurodevelopmental disorders. Maternal immune activation (MIA) during pregnancy is considered as a risk factor for schizophrenia and autism in the offspring (Brown, 2012). To study the mechanisms behind this association several animal models were developed in which pregnant rodents were infected with the influenza virus, polyinosinic:polycytidylic acid [poly (I:C)] or lipopolysaccharide (LPS) (Patterson, 2009). These models confirmed that prenatal infection leading to MIA can lead to behavioral and neurological disorders in the offspring (Shi et al., 2003; Meyer et al., 2006; Fortier et al., 2007; Lowe et al., 2008; Harvey and Boksa, 2012; Giovanoli et al., 2013; Squarzoni et al., 2014). During MIA evoked by poly (I:C), an elevated maternal serum cytokine, interleukin-6 (IL-6), was found to be critical for the development of these neurological deficits in the offspring (Samuelsson et al., 2006; Smith et al., 2007). Differences in behavioral abnormalities observed in the offspring at adult age are critically dependent on the time of maternal poly (I:C) challenge, being related to differences in cytokine responses in the fetal brain shortly after the induction of MIA (Meyer et al., 2006, 2008). However, the source of the cytokine response in the fetal brain remains a matter of debate as it can originate from maternal, placental and/or embryonic tissue. An endogenous increase in fetal brain cytokine production was demonstrated using mRNA analysis of the cytokine expression level upon maternal poly (I:C) challenge during the late gestation stage in mice (17 embryonic days, E17; Meyer et al., 2006). This was not observed when maternal poly (I:C) challenge was performed at mid gestation stage (E9; Meyer et al., 2006), a developmental age at which immature microglia, the resident immune cells of the brain, have not yet invaded the fetal central nervous system (CNS; Ginhoux et al., 2010; Rigato et al., 2011; Swinnen et al., 2013).

Microglia colonize the brain early during embryonic development (E11.5 in the mouse embryo; Ginhoux et al., 2010; Rigato et al., 2011; Swinnen et al., 2013) and are known to control several developmental processes in the brain at perinatal developmental stages (Cunningham et al., 2013; Squarzoni et al., 2014; Michell-Robinson et al., 2015). First, embryonic microglia have been shown to be involved in angiogenesis through close contact with vessel sprouts and endothelial tip cells and the secretion of soluble factors that stimulate angiogenesis during development (Fantin et al., 2010; Rymo et al., 2011). Secondly, during CNS development microglial cells clear cellular debris and induce programmed cell death in developing neurons via the production of superoxide ions (Marin-Teva et al., 2004; Wakselman et al., 2008) and tumor necrosis factor (TNF)-α (Sedel et al., 2004). Thirdly, several studies have pointed toward an important role for microglia in synaptic remodeling and synapse elimination (Tremblay et al., 2010; Paolicelli et al., 2011; Schafer et al., 2012; Zhan et al., 2014). Finally, microglial cells can also influence the development and differentiation of neural cells. Microglia-conditioned media can influence embryonic precursor migration and differentiation in primary cultures (Aarum et al., 2003; Jonakait et al., 2011). In addition, microglial cells can regulate cortical precursor proliferation and astrogenesis (Nakanishi et al., 2007). Primary culture experiments on embryonic precursor cultures showed that microglial cells are important for precursor proliferation and astrogenesis. In microglia-depleted cultures and cultures from PU.1 knock out embryos proliferation and astrogenesis were decreased. Addition of microglia to these cultures restored both processes and an abnormal increase in microglial cell numbers resulted in increased astrogenesis (Antony et al., 2011). Deactivation of embryonic microglia with tetracyclines or elimination via the macrophages suicide technique led to an increase in neural precursor cells, while microglial activation had the opposite effect (Cunningham et al., 2013).

Maternal immune activation induces an imbalance in cytokines levels, of which maternal IL-6 has been shown to be a critical mediator in inducing the effects of MIA on brain development and behavioral changes (Smith et al., 2007). IL-6 is known to induce activation of adult microglial cells; leading to the production of pro-inflammatory factors, such as nitric oxide, reactive oxygen species, proteolytic enzymes, and TNF-α by microglial cell cultures (Krady et al., 2008), microglial proliferation (*in vitro*; Streit et al., 2000) and infiltration (*in vivo*; Lacroix et al., 2002) or the upregulation of microglial CX3CR1, making them more sensitive to fractalkine signaling (Lee et al., 2010). An imbalance in cytokine levels caused by MIA might thus be able to activate embryonic microglia, even at early developmental stages, and alter their normal functions. This can trigger a cascade of events that could lead to developmental defects observed in the offspring of LPS or poly (I:C) treated pregnant mice. Indeed, MIA evoked by LPS injection evoked microglia activation and enhanced phagocytosis of neural precursors by microglia at prenatal stages in rats (Cunningham et al., 2013). However, the question remains whether an endogenous increase in fetal brain cytokine production in response to maternal poly (I:C) challenge is of microglial origin. Accordingly it remains unclear whether poly (I:C)-induced MIA results in the activation of embryonic microglia during fetal development.

To determine to what extent MIA evoked by poly (I:C) can alter cortex invasion by microglia and/or change embryonic microglial cell activation state, we evoked MIA using a single (at E11.5) or a double injection (at E11.5 and E15.5) of poly (I:C) (Meyer et al., 2006; Shi et al., 2009). This developmental time window is an important time point for cortex invasion by immature microglia as their cell density dramatically increases during this period (Swinnen et al., 2013). We show that poly (I:C)-induced MIA does not affect microglial density and activation level during embryonic development suggesting that pathological activation of embryonic microglial cells at the onset of their colonization processes cannot explain neurological deficits observed at postnatal stages in offspring after poly (I:C)-induced MIA.

## Materials and Methods

### Animals

All experiments were conducted in accordance with the European Community guiding principles on the care and use of animals and with the approval of the Ethical Committee on Animal Research of Hasselt University. Mice were maintained in the animal facility of the Hasselt University in accordance with the guidelines of the Belgian Law and the European Council Directive. To visualize microglia in the embryonic cortex the transgenic CX3CR1-eGFP knock-in mice (Jung et al., 2000) were used. The heterozygous CX3CR1-eGFP embryos used in this study were obtained by crossing wild type C57BL/6 females with homozygous CX3CR1-eGFP +/+ male mice (obtained from the European Mouse Mutant Archive – EMMA with the approval of Jung et al., 2000). The day of conception was designated as embryonic day 0.5 (E0.5).

### Maternal Immune Activation

At day E11.5 (single injection) or at E11.5 and E15.5 (double injection) mice received i.p. a dose of poly (I:C) (20 mg/kg; Polyinosinic–polycytidylic acid potassium salt; Sigma–Aldrich, Bornem, Belgium) or vehicle (saline). Five hours after injection the maternal blood was collected, the serum was aliquoted and stored at -80°C until the IL-6 assay was performed (Shi et al., 2003; Smith et al., 2007). The maternal IL-6 concentrations were determined using the Mouse IL-6 ELISA Kit from Thermo Scientific (Rockford, IL, USA), following the manufacturer’s instructions. The analysis was conducted using a FLUOstar OPTIMA plate reader (BMG Labtech, Ortenberg, Germany).

### Fluorescent Immunostaining of Embryonic Brains

Pregnant mice were sacrificed and embryonic tissue processed as described before (Swinnen et al., 2013). The heads of E11.5 and E12.5 embryos were fixed in 4% paraformaldehyde for 3 h at 4°C and 5 h for E17.5 embryos. After fixation, the embryonic heads were cryoprotected overnight in phosphate-buffered saline (PBS) + 30% sucrose, frozen in optimal cutting temperature compound (Tissue-Tek) and stored at -80°C until sectioned. Ten micrometer-thick coronal tissue sections were cut on a Leica CM1900 uv cryostat, mounted on Superfrost Plus glasses and stored at -20°C until staining.

To check whether embryonic microglia can be directly activated by poly (I:C), IL-6 or LPS, 300-μm thick coronal brain slices (E15.5) were cultured for 24 h with either saline, poly (I:C) (50 μg/ml), IL-6 (10 ng/ml), or LPS (1 μg/ml). To this end, pregnant mothers were euthanized at E15.5. Embryonic brains were isolated in ice-cold PBS-glucose (pH 7.4; 25 mM), embedded in 3% low melting agarose (Fisher Scientific) and sliced coronally at a thickness of 300 μm using a Microm HM650V Vibrating Blade Microtome. Slices were mounted on MilliCell organotypic inserts (Millipore) and maintained in semi-hydrous conditions at 37°C and 5% CO_2_ for 24 h. The media consisted of Neurobasal medium supplemented with 2 mM L-glutamine, B27 supplement, N2 supplement, and 0.5% penicillin–streptomycin (all from Invitrogen) with either saline, poly (I:C) (50 μg/ml), IL-6 (10 ng/ml) or LPS (1 μg/ml) added. Afterward slices were fixed for 1 h in 4% PFA and cryoprotected overnight in PBS + 30% sucrose, frozen in optimal cutting temperature compound (Tissue-Tek) and stored at -80°C until sectioned. Ten micrometer-thick coronal tissue sections were cut on a Leica CM1900 uv cryostat, mounted on Superfrost Plus glasses and stored at -20°C until staining.

In order to determine the activation state of the microglia, we used antibodies against interleukin (IL)-1β, inducible nitric oxide synthase (iNOS) and Mac-2/Galectin-3 (Rigato et al., 2011; Cunningham et al., 2013). All primary antibodies and working solutions are listed in **Table 1**.

**Table 1:** Overview of the antibodies used for immunostainings and flow cytometry experiments.

Antibody	Company	Reference	Dilution
**Immunohistochemistry**			
Anti-IL1β (rabbit polycl.)	Abcam	ab9722	1:100
Anti-iNOS (rabbit polycl.)	Abcam	ab15323	1:250
Anti-Mac-2 (rat monocl.)	American type culture collection	TIB-166	1:250
**Flow cytometry**			
Anti-IL1β PE (rat monocl.)	LifeSpan BioSciences	LS-C184791	1:300
Anti-iNOS PE-Cy7 (rat monocl.)	eBioscience	25-5920	1:300
Anti-Mac-2 PE (rat monocl.)	eBioscience	12-5301	1:300

### Isolation of Microglia and Flow Cytometry Experiments

Brains were isolated from CX3CR1-eGFP E17.5 embryos from mothers subjected to a single saline or poly (I:C) injection on E11.5, or a double poly (I:C) injection on E11.5 and E15.5. All steps occurred at 4°C or on ice, unless stated otherwise, to avoid microglia activation. Meninges were removed, the cortical area identical to the immunohistochemical analysis was dissected out and incubated during 30 min at 30°C in DMEM/F-12(1:1) + GlutaMAX (Life Technologies) containing 48 U/ml Papain from papaya latex (Sigma). Papain containing supernatants was discarded and the tissue was mechanically disrupted in medium through fast pipetting using a 1 ml pipet. Afterward, the homogenate was centrifuged at 400*g* during 5 min, resuspended in 40% isotonic Percoll (GE Healthcare) and centrifuged at 700*g* during 10 min without break. The pellet was resuspended in PBS and filtered through a 35 μm cell strainer. Cell suspensions were fixed and permeablized in Cytofix/Cytoperm buffer (BD Cytofix/Cytoperm^TM^ Plus Fixation/Permeabilization Kit, BD Biosciences) during 20 min on ice, washed and incubated on ice for 30 min in Perm/Wash buffer with a mix of fluorochrome-conjugated rat anti-mouse antibodies: iNOS-PE-Cy7 (clone CXNFT, eBioscience), Mac-2-PE (clone eBioM3/38, eBioscience) and, IL1β-PE (clone 11n92, LifeSpan BioSciences) (**Table 1**). The following isotype controls were used: Rat IgG2aκ PE-Cy7, Rat IgG2aκ PE and Rat IgG2b PE (all from eBioscience). After washes, cells were resuspended in FACS buffer (PBS, 2% FCS, sodium azide), acquired in a FACS Aria II and analyzed with FACS Diva 6.1.3 software (BD Biosciences). Isotype-marker overlay graphs were created in FlowJo 10.0.8 Software. Inside the singlet population, the eGFP positive microglia (1000–12000 cells per experiment) were gated (**Figure 5A**), and within this population, the percentage of Mac-2, iNOS, and IL1β positive microglia was analyzed. Isotype controls were used to gate the positive cell population (**Figure 5B**). Per group, embryos were derived from one to three different mothers (saline, single poly (I:C), double poly(I:C)). BV-2 cells (Supplementary Data) were used as positive controls for the different antibodies (Supplementary Figure S1).

### Analysis and Statistics

Quantitative analysis of microglial cells was performed on images of coronal embryonic brain sections. We focused our analysis on the cerebral cortex area located dorsally to the lateral ganglionic eminences (LGE) and medial ganglionic eminences (MGE), containing the frontal and pariental cortex on E11.5 and E12.5, and the somatosensory and motor cortex at E17.5. This region of the cortex is well characterized on the functional and cellular level and the two GE structures are the major sources of cortical interneurons during embryonic neurogenesis (Tan et al., 1998; Anderson et al., 2001). For the quantifications of the hippocampal area at E17.5 only the dorsal hippocampus was included in the analysis.

Images were taken with a Nikon Eclipse 80i microscope and a Nikon digital sight camera DS-2MBWc [10x Nikon plan objective (numerical aperture (NA) of 0.25) and a 20x Plan Fluor objective (NA of 0.5)]. Images (1600 × 1200) were analyzed with ImageJ 1.45e software (NIH, USA; http://rsb.info.nih.gov/ij/). Only eGFP-positive cell bodies were taken into account for the measurements. Density analysis was performed by counting the number of eGFP positive cell bodies per mm^2^ (Swinnen et al., 2013). For analysis of activation state we calculated the percentage of the eGFP positive cells that were also showing immunoreactivity for the activation marker. All values are expressed as mean ± SEM. The number of sections used is indicated as *n*, the number of embryos or blood samples as *N*; # sections/# embryos is thus designated in the text as *n/N*. Statistical significance was assessed by non-parametric Mann–Whitney test or Kruskal–Wallis test, *P*-values smaller than 0.05 were considered significant.

## Results

An increase in IL-6 level in the maternal blood is a crucial factor in the development of MIA-induced deficits and changes observed in the offspring (Smith et al., 2007). To control that the poly (I:C) injection procedure we used evoked an increase in IL-6 level in the maternal blood, we analyzed the IL-6 level in the maternal serum samples 5 hours after injection of either saline or poly (I:C). We found a significant increase (*P* < 0.0001; Mann–Whitney test) in the level of IL-6 in the sera of female mice primed with poly (I:C) (1876 ± 389.2 pg/ml, *N* = 22) when compared to those injected with saline (14.8 ± 3.3 pg/ml, *N* = 26), thus indicating that the mice in the poly (I:C) group effectively suffered from a systemic immune response.

In response to brain injury, microglia proliferate and shift to beneficial or detrimental activation states depending on the local environment. When activated, microglial cells adopt a phagocytic phenotype in order to clear dying cells (Kettenmann et al., 2011). In pathological conditions, such as in the mouse model of LPS-induced MIA, phagocytosis of neuronal precursor cells by microglia was also increased, which resulted in a decrease in the size of the precursor cell pool in the cerebral cortex (Cunningham et al., 2013). It must also be noted that microglial disturbances were also observed in patients suffering from autistic or schizophrenic disorders. Microglial activation has been observed in the brains of autistic (Vargas et al., 2005; Morgan et al., 2010) and schizophrenic patients (Radewicz et al., 2000; Wierzba-Bobrowicz et al., 2005; Monji et al., 2013). Recent studies also indicated that there is an increase in microglial density in different brain regions in the adult poly (I:C) MIA offspring (Juckel et al., 2011; Ratnayake et al., 2012).

**FIGURE 1:**
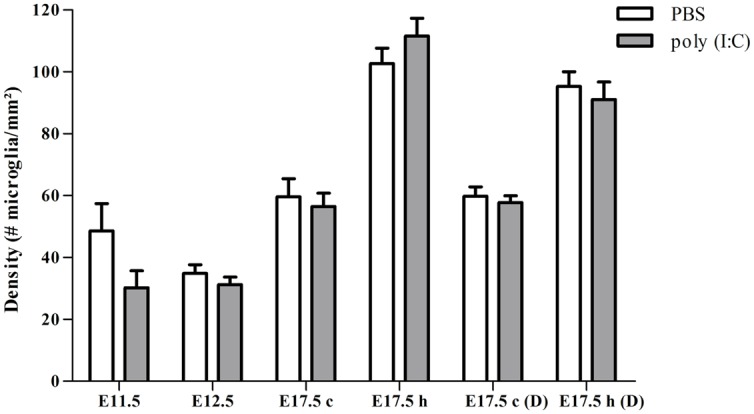
**Embryonic microglial cell density is not increased after single and double injection of poly (I:C).** Microglial cell density in the cortex and hippocampal area was not affected after poly (I:C)-induced MIA. Values are mean ± SEM of the number of microglial cells per mm^2^, Mann–Whitney test was used for statistical analysis. When injected at E11.5 the numbers of embryonic brains in the saline and poly (I:C) group were, respectively: E11.5 = 4/5; E12.5 = 12/7; E17.5 cortex = 6/8; E17.5 hippocampus = 5/8. When injected at E11.5 and E15.5 numbers of embryonic brains in the saline and poly (I:C) group were, respectively: E17.5 cortex = 5/6; E17.5 hippocampus = 6/6. c, cortex; h, hippocampal area; D, double injection.

To determine if poly (I:C)-evoked MIA alters the embryonic microglial cell colonization process in the fetal brain we compared cell density after single injection of poly (I:C), double injection of poly (I:C) or saline treatments, in the cortex at E11.5, E12.5 and at E17.5 (single injection) or at E17.5 (double injections) and in the hippocampal area at E17.5 (single and double injections). At all ages tested we did not find any significant difference in microglia cell density (Mann–Whitney test; *P* > 0.05, for detailed *P*-values see **Table 2**) in the cortex or in the hippocampus after a single or after double injections (**Figure 1**; **Table 2**), thus suggesting that poly (I:C)-evoked MIA does not alter early invasion of the cortex and the hippocampus by microglial cells in the embryo.

**Table 2:** Microglial cell density in the cortex and hippocampal area of embryos derived from the control group and the group that was subjected to maternal inflammation at E11.5 or at E11.5 and E15.5.

Brain structure	Cortex			Hippocampus
**Embryonic age**	**E11.5**	**E12.5**	**E17.5**	**E17.5**

**Single injection at E11.5**			
Saline	48.6 ± 8.8	34.9 ± 2.8	59.6 ± 5.8	122.5 ± 4.9
Poly (I:C)	32.2 ± 5.7	37.8 ± 2.9	56.5 ± 4.3	111.6 ± 5.7
*P* value	0.191	0.375	0.573	0.435

**Brain structure**	**Cortex**			**Hippocampus**

**Embryonic age**	**E17.5**			**E17.5**

**Double injection at E11.5 and E15.5**			
Saline	59.8 ± 3.1			95.3 ± 4.8
Poly (I:C)	57.8 ± 2.2			91.0 ± 5.7
*P* value	0.931			0.699

*Values are mean ± SEM of the number of microglial cells per mm^2^, Mann–Whitney test was used for statistical analysis. When injected at E11.5 the numbers of embryonic brains in the saline and poly (I:C) group were, respectively: E11.5 = 4/5; E12.5 = 12/7; E17.5 cortex = 6/8; E17.5 hippocampus = 5/8. When injected at E11.5 and E15.5 numbers of embryonic brains in the saline and poly (I:C) group were, respectively: E17.5 cortex = 5/6; E17.5 hippocampus = 6/6.*

**FIGURE 2:**
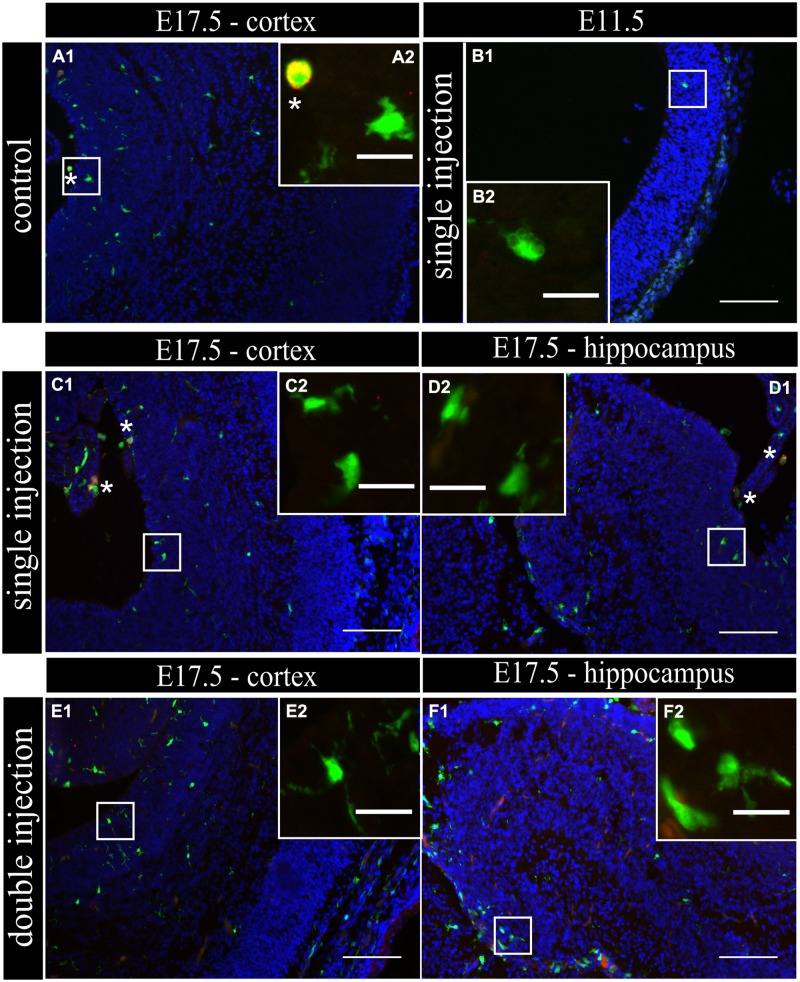
**Embryonic microglial cell population is poorly immunoreactive to the Mac-2/Galectin-3 antibody after single and double injection of poly (I:C). (A–F1)** Coronal sections of embryonic brains, with cell nucleus staining in blue (DAPI) and microglial (CX3CR1-eGFP) cells in green. Immunohistochemical staining using a Mac-2 antibody (red) showed that at E17.5 almost no microglial cells in the cortex were immunoreactive for Mac-2 **(A2)** after injection with saline. At E11.5 **(B2)** and E17.5 **(C2,E2)** in the cortex and E17.5 hippocampal area **(D2,F2)** there was no increased percentage of microglial cells expressing the activation marker after poly (I:C) challenge compared to control. White square indicates the location of the cells in the tissue showed in the inset; ^∗^ indicates a Mac-2 positive eGFP cell. Examples of one control brain area and poly (I:C) group only as they were not significantly different. Scale bar = 100 μm and for insets = 20 μm.

**FIGURE 3:**
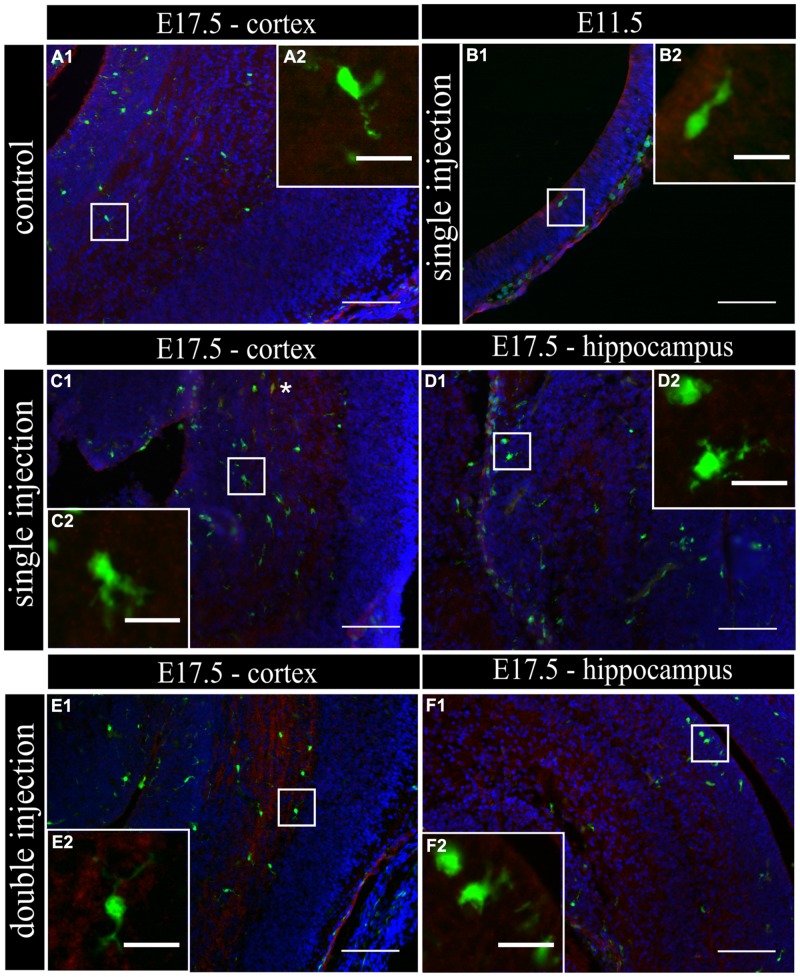
**Embryonic microglia show no increased expression of IL1β after single and double injection of poly (I:C). (A–F1)** Coronal sections of embryonic brains, with cell nucleus staining in blue (DAPI) and microglial (CX3CR1-eGFP) cells in green. Immunohistochemical staining using an IL1β antibody (red) showed that at E17.5 almost no microglial cells in the cortex were immunoreactive for IL1β **(A2)** after injection with saline. At E11.5 **(B2)** and E17.5 **(C2,E2)** in the cortex and E17.5 hippocampal area **(D2,F2)** there was no increased percentage of microglial cells expressing the activation marker after poly (I:C) challenge compared to control. White square indicates the location of the cells in the tissue showed in the inset; ^∗^ indicates an IL1β positive eGFP cell. Examples of one control brain area and poly (I:C) group only as they were not significantly different. Scale bar = 100 μm and for insets = 20 μm.

**FIGURE 4:**
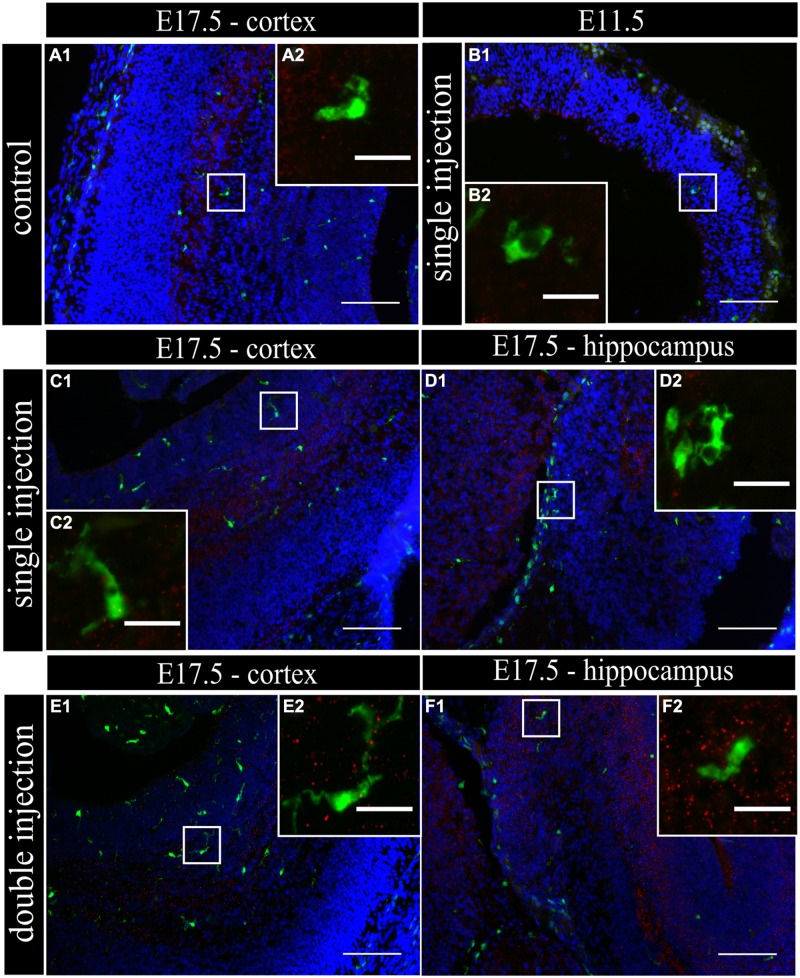
**Embryonic microglia cell population is poorly immunoreactive to the iNOS antibody after single and double injection of poly (I:C). (A–F1)** Coronal sections of embryonic brains, with cell nucleus staining in blue (DAPI) and microglial (CX3CR1-eGFP) cells in green. Immunohistochemical staining using an iNOS antibody (red) showed that at E17.5 almost no microglial cells in the cortex were immunoreactive for iNOS **(A2)** after injection with saline. At E11.5 **(B2)** and E17.5 **(C2,E2)** in the cortex and E17.5 hippocampal area **(D2,F2)** there was no increased percentage of microglial cells expressing the activation marker after poly (I:C) challenge compared to control. White square indicates the location of the cells in the tissue showed in the inset. Examples of one control brain area and poly (I:C) group only as they were not significantly different. Scale bar = 100 μm and for insets = 20 μm.

To determine if MIA induced a change in microglial activation level after a single poly (I:C) injection (E11.5), we performed an immunostaining for three different activation markers: Mac-2/Galectin-3, iNOS and IL1β at E11.5 and E17.5. Mac-2/Galectin-3 is a marker of microglial phagocytic activation state (Dumic et al., 2006; Rotshenker, 2009) while iNOS and IL1β are markers of a cytotoxic activation state (Cunningham et al., 2013). At E11.5 none of the microglia located in the cortex was immmunopositive for Mac-2 staining both after saline injection (*n/N* = 14/3) and after poly (I:C) challenge (*n/N* = 18/3; **Figure 2B1,B2**). At E17.5, 2.5 ± 0.5% (*n/N* = 38/4) of the microglia in the cortex (**Figure 2A1,A2**) and 3.2 ± 0.7% (*n/N* = 27/4) of the microglia in the hippocampal area expressed Mac-2 after saline injection. We did not find any significant difference (Kruskal–Wallis test; *P* = 0.448) after poly (I:C) challenge. After poly (I:C) challenge, 1.9 ± 0.7% (*n/N* = 23/4) of the microglia in the cortex and 2.5 ± 1% (*n/N* = 15/4) of microglia in hippocampal area expressed Mac-2 (**Figures 2C1,C2,D1,D2**). We next investigated the expression of IL1β and iNOS (Cunningham et al., 2013) to determine if embryonic microglia can adopt a cytotoxic activation state after a single injection of poly (I:C). Induction of MIA by a single injection of poly (I:C) did not result in a significant increase in the percentage of microglia expressing IL1β either at E11.5 and E17.5 (Kruskal–Wallis test; *P* = 0.136). In control conditions, 0 ± 0% (*n/N* = 6/3) and 2.2 ± 1% (*n/N* = 15/4) of microglia located in the cortex expressed IL1β at E11.5 and E17.5 (**Figure 3A1,A2**), respectively, while 3.1 ± 1.3% (*n/N* = 17/4) expressed IL1β in the hippocampal area (E17.5). After poly (I:C) challenge, 3.3 ± 3.3% (*n/N* = 10/3) and 3.5 ± 1% (*n/N* = 19/4) of microglia located in the cortex expressed IL1β at E11.5 (**Figure 3B1,B2**) and at E17.5 (**Figure 3C1,C2**), respectively, while 7.2 ± 2.6% (*n/N* = 17/4) expressed IL1β in the hippocampal area (E17.5; **Figure 3D1,D2**). We found similar results when analyzing iNOS expression at E11.5 and E17.5 in the cortex and in the hippocampal area (E17.5). Cortical iNOS expression in control conditions [E11.5: 8.3 ± 5.7%, *n/N* = 10/3; E17.5: 2.0 ± 1.1%, *n/N* = 15/4 (**Figure 4A1,A2**)] was not significantly different when compared to the poly (I:C) condition [E11.5: 0 ± 0%, *n/N* = 8/3 (**Figure 4B1,B2**); E17.5: 1.9 ± 1.1%, *n/N* = 12/4 (**Figure 4C1,C2**; Kruskal–Wallis test; *P* = 0.471)]. In the hippocampal area, 1.5 ± 1.0% of microglia (*n/N* = 14/4) express iNOS in control conditions while 0 ± 0%, of microglia (*n/N* = 10/4) express iNOS after poly (I:C) challenge (**Figure 4D1,D2**, being not significantly different (Kruskal–Wallis test; *P* = 0.471)).

This lack of change in embryonic microglia activation state after a single poly (I:C) injection could possibly lead only to a “primed” microglial state. Indeed, two injections of LPS were necessary in rat to elicit MIA induced microglia dysfunction during phagocytosis of cortical neural precursor cells (Cunningham et al., 2013), suggesting that the microglial phenotype could become only fully altered after the second inflammatory challenge. To determine if this is also the case for poly (I:C) we reanalyzed microglial density and activation level after a repeated injection of poly (I:C). Consequently, the mothers suffered from a double immune stimulation (on E11.5 as well as on E15.5). Despite the presence of a maternal immune response after both injections, there was no significant increase in microglial cell density (Mann–Whitney test; *P* > 0.05, for detailed *P*-values see **Table 2**) (**Figure 1**; **Table 2**). Microglial activation states were analyzed at E17.5 as described above. We did not find any significant difference (Kruskal–Wallis test; Mac-2, *P* = 0.139; IL1β, *P* = 0.945; iNOS, *P* = 0.093) in the percentage of microglia expressing Mac-2, IL1β, or iNOS between control conditions and after double injections of poly (I:C). After double injections of poly (I:C) the percentage of microglia immunoreactive for Mac-2 antibody was 0 ± 0% (*n/N* = 29/6) in the cortex (**Figure 2E1,E2**) and 2.0 ± 0.7% (*n/N* = 22/6) in the hippocampal area (**Figure 2F1,F2**). In the cortex (**Figure 3E1,E2**) and hippocampal area (**Figure 3F1,F2**) 1.4 ± 0.7% (*n/N* = 34/6) and 1.4 ± 1.0% (*n/N* = 25/6) of the microglial cells showed immunoreactivity for the IL1β antibody, while 1.8 ± 0.7% (*n/N* = 34/6) and 0 ± 0% (*n/N* = 23/6) of the microglia were positive for iNOS in the cortex (**Figure 4E1,E2**) and hippocampal area (**Figure 4F1,F2**), respectively. These results indicate that even double injections of poly (I:C) did not evoke microglia activation in the embryo.

**FIGURE 5:**
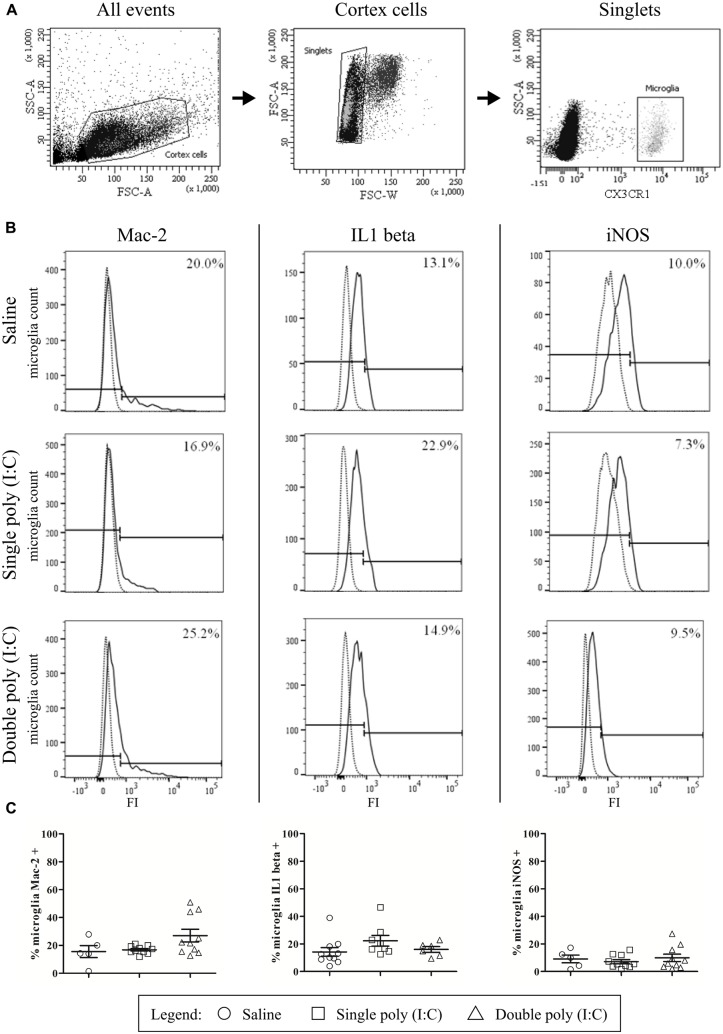
**Flow cytometry reveals that embryonic microglial cells show a poor expression of activation markers Mac-2, IL1β and iNOS. (A)** Gating strategies for the microglial cells. In the whole embryonic cortex cell suspension, a gate was created on the non-debris population (left). Inside this population, single cells were selected (middle) and within this population, the microglial cells were gated based on CX3CR1-eGFP intensity (right). SSC, Side scatter; FSC, Forward scatter. **(B)** Gating strategies for positive Mac-2, iNOS and IL1β populations. Microglial cell count of representative samples is shown for Mac-2 (left), IL1β (middle) and iNOS (right; full lines) for embryos derived from saline, single poly (I:C) and double poly (I:C) injected mothers. Gates for positive populations were drawn based on the isotype fluorescence intensity (dotted lines). FI, fluorescence intensity. **(C)** Left panels: at E17.5 only a small percentage of microglial cells shows reactivity for Mac-2. There is no significant effect of poly (I:C) injection on this percentage. Number of embryos tested: Saline *N* = 5; single poly (I:C) *N* = 10 and double poly (I:C) *N* = 10. Middle panels: in control conditions, less than 15% of the microglial cells is positive for IL1β. There is no significant effect of poly (I:C) injection on this proportion. Number of embryos tested: Saline *N* = 10; single poly (I:C) *N* = 8 and double poly (I:C) *N* = 6. Right panels: at E17.5 less than 10% of the microglial cells is positive for iNOS. Poly (I:C) challenge has no significant effect on this percentage. Number of embryos tested: saline *N* = 5; single poly (I:C) *N* = 10 and double poly (I:C) *N* = 10.

In addition to the immunohistochemical stainings, the presence of the activation markers on microglial cells at E17.5 was investigated by flow cytometry. The gating strategy and positive controls are shown in **Figures 5A,B** and Supplementary Figure S1. The results of the flow cytometric quantifications were similar to those obtained by immunohistochemistry. There was no significant difference in the proportion of microglial cells that were positive for Mac-2 after single poly (I:C) injection (16.8 ± 0.0%; N = 10) or double poly (I:C) injection (27.0 ± 4.6%; *N* = 10) when compared to the control group (15.5 ± 4.3; *N* = 5; **Figure 5C**, left; Kruskal–Wallis test, *P* = 0.161). The proportion of microglial cells that were positive for IL1β in the control group (14.2 ± 3.1%; *N* = 10) was not significantly different (**Figure 5C**, middle; Kruskal–Wallis test, *P* = 0.093) to the percentage of microglia that was positive for IL1β after a single (22.3 ± 3.9%; *N* = 8) or double poly (I:C) injection (16.0 ± 2.1%; *N* = 6). The percentage of microglial cells positive for iNOS in the control group was 9.1 ± 2.8% (*N* = 5). There was no significant effect (**Figure 5C**, right; Kruskal–Wallis test, *P* = 0.816) of a single poly (I:C) (7.1 ± 1.5%; *N* = 10) or double poly (I:C) challenge (9.9 ± 2.7%; *N* = 10) on the percentage of microglia expressing this marker.

**FIGURE 6:**
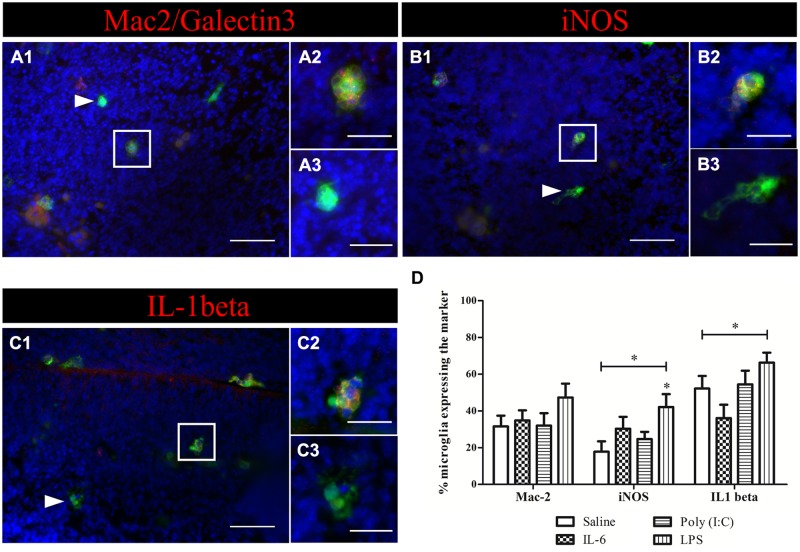
**Microglial activation in acute brain slices.** Example of activation marker stainings on acute slices treated with LPS. **(A)** Immunohistochemical staining for Mac-2/Galectin-3 (red), nuclei were visualized with DAPI (blue; **A1)**. Microglia (green) positive (**A1** white square, **A2**) for Mac-2/Galectin-3 (red) and microglia that do not express the marker (white triangle, **A3**) were present in the slice. **(B)** Immunohistochemical staining for iNOS (red), nuclei were visualized with DAPI (blue; **B1)**. Microglial cells that were positive (white square **B1,B2**) and negative (white triangle **B1,B3**) for iNOS (red) were observed in the slice after LPS treatment. **(C)** Immunohistochemical staining for IL1β (red), nuclei were visualized with DAPI (blue; **C1)**. Microglial cells that were positive (white square **C1,C2**) and negative (white triangle **C1,C3**) for IL1β (red) were observed in the slice after LPS treatment. Examples of the different immunostainings were taken from slices treated for 24 h with 1 μg/ml LPS. Scale bar = 50 μm and for inserts = 20 μm. White squares indicate the microglia positive for the marker and shown in higher magnification **(A–C2)**, white triangles indicate microglia negative for the marker and shown in higher magnification **(A–C3)**. **(D)** Quantification of the expression of three activation markers (Mac-2, iNOS, and IL1β) by microglia in E15.5 brain slices cultured for 24 h with IL-6 (10 ng/ml), poly (I:C) (50 μg/ml), or LPS (1 μg/ml). Kruskal–Wallis test was used for statistical analysis. Number of treated slices in control and IL-6 group *N* = 4; LPS and poly (I:C) group *N* = 5. Number of cryosections for Mac-2/iNOS/IL1β in: saline group *n* = 23/23/27; IL-6 group *n* = 22/19/16; poly (I:C) group *n* = 18/25/19; LPS group *n* = 21/21/22 (all derived from three different embryos). (^∗^*p* < 0.05).

The absence of activation marker expression by microglia after poly (I:C) challenge raised the question whether fetal microglia can be directly activated by a poly (I:C) challenge as suspected for LPS (Cunningham et al., 2013) and IL-6 (Smith et al., 2007). To address this issue we analyzed the activation state of microglia in acute embryonic brain slices (E15.5) after exposure to IL-6, poly (I:C) or LPS. The percentage of microglial cells expressing Mac-2/Galectin-3, iNOS, and IL1β were analyzed 24 h after immune challenge of the slices (**Figure 6D**). **Figure 6** insets show examples of microglial cells that did (**Figures 6A–C2**) or did not show immunoreactivity (**Figures 6A–C3**) for the activation markers tested (Mac-2, IL1β, and iNOS). In control conditions 31 ± 5.9%, (*n/N* = 23/4) of microglia were immunoreactive for Mac-2 antibody. This percentage was significantly higher (Kruskal–Wallis test; *P* < 0.0001) than that observed *in vivo* indicating that an *in vitro* environment promotes microglia phagocytic activation state. However, there was no significant effect (Kruskal–Wallis test; *P* = 0.274) of IL-6, poly (I:C) or LPS treatment on the percentage of microglia being immunoreactive to Mac-2 antibody (**Figure 6D**), being 34 ± 5.5% (*n/N* = 22/4) after IL-6 exposure, 32 ± 6.7%, (*n/N* = 18/5) after poly (I:C) exposure and 47 ± 7.5% (*n/N* = 21/5) after LPS exposure (**Figure 6D**). As observed for Mac-2, the percentage of IL1β immunoreactive microglia was significantly higher than in *in vivo* conditions [in control conditions 52 ± 6.8%, (*n/N* = 27/4; Kruskal–Wallis test; *P* < 0.0001)] and for iNOS a trend to a higher percentage was observed under control conditions [in control conditions 18 ± 5.7%, (*n/N* = 23/4; Kruskal–Wallis test; *P* = 0.091)]. As shown in **Figure 6D** treatment with IL-6 or poly (I:C) did not significantly change the percentage of microglia immunoreactive for IL1β or iNOS antibodies. When looking at IL1β immunoreactivity, 36 ± 7.2% (*n/N* = 16/4) of the microglia was positive after IL-6 exposure and 54 ± 7.5%, (*n/N* = 19/5) after poly (I:C) exposure (**Figure 6D**). For iNOS they were 30 ± 6.5% (*n/N* = 19/4) after IL-6 exposure and 25 ± 3.9%, (*n/N* = 25/5) after poly (I:C) exposure (**Figure 6D**). However, we found that LPS, contrary to IL-6 or poly (I:C), can directly activate microglia to a detrimental activation state. Indeed LPS exposure significantly increased the percentage of microglia immunoreactive for IL1β (Kruskal–Wallis test; *P* = 0.025) or iNOS antibodies (Kruskal–Wallis test; *P* = 0.025). In the presence of LPS 66 ± 5.5 (*n/N* = 22/5) and 42 ± 7.1% (*n/N* = 21/5) of microglia were immunoreactive for IL1β antibody or iNOS antibody, respectively.

## Discussion

Maternal immune activation-induced behavioral and neurological alterations observed in the offspring at juvenile and adult stages in animals are supposed to be correlated with the etiology of neuropsychiatric disorders in humans. Our study in mice demonstrates, for the first time, that MIA evoked by single or double poly (I:C) injections does not change microglia density and their activation state in the embryo *in vivo.* This suggests that the behavioral and neurological alterations in the offspring cannot be related to the alteration of the activation state of embryonic microglial cells. Our *in vitro* studies indicated that microglia cannot be directly activated by poly (I:C) or IL-6 exposure, contrary to the activation observed upon LPS application.

Several observations suggest that the different infectious triggers induce differences in activation of embryonic microglia. The cytokine IL-6 can cross the placenta barrier *in vivo* when maternal inflammation was induced during mid-gestation (Kohmura et al., 2000; Ashdown et al., 2006; Dahlgren et al., 2006), but it is not clear whether poly (I:C) as well can cross the placenta (Brown and Patterson, 2011). LPS is shown to cross the placenta barrier *in vivo* when maternal inflammation was induced during early gestation (Cai et al., 2000; Kohmura et al., 2000), but this was not the case when LPS was injected at late gestation (Ashdown et al., 2006). Although extrapolation of these results to a poly (I:C) challenge would suggest that embryonic microglia are directly or indirectly activated in response to poly (I:C)-induced MIA at mid gestation, we could not find any evidence for microglia activation in this study. Previously, microglia dysfunction observed after poly (I:C)-induced MIA was only reported in offspring at postnatal and adult age (Juckel et al., 2011; Manitz et al., 2012). In that way it is of interest to compare in parallel the effect of MIA induced by different infectious agents on the embryonic microglia. Studies using single or repeated LPS challenge showed that this leads to microglial activation: in the fetal sheep brain, microglial cell numbers increased as well as the number of activated/amoeboid cells (Mallard et al., 2003; Hutton et al., 2008; Kuypers et al., 2013); in the rat embryo the percentage of microglia expressing iNOS and IL1β was increased (Cunningham et al., 2013) and postnatally a changed immunoreactivity by microglial cells was still observed (Cai et al., 2000); and in mice Iba-1 reactivity was increased during late embryonic and early postnatal stages (Le Belle et al., 2014). In conclusion, the time of injection and the nature of the infectious trigger determine whether an activation of the embryonic microglia does or does not participate to developmental neurological defects observed in MIA offspring (Garay et al., 2012). In addition the microglial response might be species dependent. However, a thorough comparison of the effect of MIA in different species is difficult to make for several reasons. For example, some studies use the mRNA and/or protein expression level of different cytokines as read-out (Garay et al., 2012) while others use immunohistochemistry (Cunningham et al., 2013; Giovanoli et al., 2013) or cell number (Hutton et al., 2008; Manitz et al., 2012) to investigate microglial cell activation after MIA. In addition, the effect of MIA is studied on several different postnatal and adult time points.

Microglial activation in postnatal to adult brains has been found to correlate to neurodevelopmental diseases. An active neuroinflammatory process, with microglial cell activation, was described in the brains of autistic patients (Vargas et al., 2005; Morgan et al., 2010) and of schizophrenic patients (Radewicz et al., 2000; Wierzba-Bobrowicz et al., 2005; Monji et al., 2013). However, it remains unclear if microglia activation participates to neuronal disorders or reflects a normal microglia response to neural dysfunctions. Our results show that poly (I:C)-induced MIA does not lead to activation of embryonic microglia. Yet, they cannot exclude that the embryonic microglial cells become primed, which could result in a more vigorous response to a subsequent inflammatory stimulation in the adult. In some neurodegenerative disease models in rodents (for example Alzheimer’s, Parkinson’s, and prion disease) the injection of LPS or poly (I:C) leads to a more severe pathology. The combined exposure of a prenatal immune challenge [poly (I:C) at E9] and peripubertal stress (from P30 to 40) resulted in the development of sensorimotor gating deficiencies and led to increased dopamine levels in the adult hippocampus (Giovanoli et al., 2013). At peripubertal age, the combination of both stressors resulted in altered neuroimmune responses, presented as increased microglial cell number and elevated levels of IL1β and TNFα in the hippocampus and prefrontal cortex (Giovanoli et al., 2013). These latter changes were transient, as they were not longer present in the adult. Finally, low doses of poly (I:C) worsened the deficits in pre-pulse inhibition and latent inhibition in 16 week-old mice with mutations in a schizophrenia susceptibility gene but had no effect in wild-type animals, thus indicating that genetic and environmental factors can interact to worsen the schizophrenia-related behavior (Lipina et al., 2013).

MIA induces not only a cytokine response in the maternal unit but also alters several cytokine levels in the placenta and in the fetus (Patterson et al., 2008; Pratt et al., 2013). Under normal conditions cytokines are present in the placental unit where they play an important role in controlling the tissue homeostasis and balance of the different T-cell types present in this structure. In addition, toll-like receptors (TLR), such as TLR-2 and 4, are expressed on human chorionic villi (Jonakait, 2007). Maternal injection with IL-6 is known lead to endocrine changes in the placenta (Hsiao and Patterson, 2011) and injection of a high dose of LPS results in placental inflammation (Girard et al., 2010) and induction of pro-inflammatory cytokines in the amniotic fluid (Gayle et al., 2004). In addition, a direct injection of LPS into the uteroplacental circulation leads to a reaction in the embryonic brain, suggesting the placental unit can contribute to perinatal brain damage through the induction of an inflammatory reaction as a response to infection during pregnancy (Hutton et al., 2008). This complicates elucidating the site where the cytokines act upon to potentially alter brain development since they can act directly on neural progenitors and neurons (Bauer et al., 2007; Deverman and Patterson, 2009). For example, IL-6 and LIF can influence the differentiation of neural progenitor cells (Nakanishi et al., 2007).

These data, in combination with the lack of microglial activation in our MIA study suggests that the acute maternal inflammation induced by poly (I:C) could affect other systems or cell types during embryonic stages. These MIA-induced early abnormalities might result in an altered CNS environment in the offspring that in turn affects the microglial cells at later developmental stages. This hypothesis is supported by the observed changes in neurotransmitter systems in the adult offspring and not in the pre-pubertal period after challenge with poly (I:C) (Manitz et al., 2012). GABAergic gene expression, like GABA receptor subunits and vesicular transporters, can be altered in the adult prefrontal cortex after MIA (Richetto et al., 2013). In addition, serotonin and glutamate signaling was altered (Holloway et al., 2013). These changes were not present at pre-pubertal ages. It is also important to note that, although microglia do not invade the CNS of mouse embryo at E9 (Rigato et al., 2011; Swinnen et al., 2013), poly (I:C) challenge at this gestation stage resulted in the suppression of spatial exploration in the adult (Meyer et al., 2006). This reinforces the idea that embryonic microglia dysfunction, if any, is unlikely to be the main mechanism inducing developmental disorders featuring pathological behavior. Accordingly, poly (I:C) challenge at E9 did not evoke any increase in cytokine mRNA level in the fetal brain (Meyer et al., 2006). Poly (I:C) might induce developmental deficits via direct action on neuronal development. However, our results cannot exclude that poly (I:C) evokes an embryonic microglia priming resulting in an exaggerated response of microglia to homeostatic disturbances at postnatal stages and subsequently makes neuronal dysfunction worse.

## Conclusion

Our findings show that a single and double injection of poly (I:C) is not sufficient to induce changes in fetal microglia activation phenotype during mid or late embryonic development. In addition they suggest a different response of the embryonic brain to MIA depending on the challenge procedure used.

## Author Contributions

Induction of MIA, IL-6 ELISA assays, immunohistochemical stainings, and quantifications were done by SS, SMTS, and NS. Guidance of the study, writing, and correction of the manuscript was performed by all authors.

## Conflict of Interest Statement

The authors declare that the research was conducted in the absence of any commercial or financial relationships that could be construed as a potential conflict of interest.
