# Supplementary material for: Maternal immune activation evoked by polyinosinic:polycytidylic acid does not evoke microglial cell activation in the embryo
Source: Front Cell Neurosci. 2015 Aug 5;9:301. doi: 10.3389/fncel.2015.00301 (PMC4525016; doi:10.3389/fncel.2015.00301)
Supplement: Supplementary file 1 [file Data_Sheet_1.DOCX]

***Supplementary Material***

**Maternal immune activation evoked by polyinosinic:polycytidylic acid does not evoke microglial cell activation in the embryo.**

**Silke Smolders, Sophie MT Smolders, Nina Swinnen, Annette Gärtner, Jean-Michel Rigo, Pascal Legendre and Bert Brône ^*^**

*** Correspondence:** Corresponding Author: bert.brone@uhasselt.be

1. **Supplementary Material and Methods**
   1. **BV-2 cell culture and flow cytometry**

The immortalized mouse microglial cell line BV-2 (kindly provided by Dr. F. Stassen, Maastricht, The Netherlands) was cultured in Dulbecco's Modified Eagle Medium containing 10% fetal calf serum, 2mM glutamine and 1 % penicillin streptomycin (all from Life Technologies). Cells were detached by incubation with PBS-EDTA 20 mM for 10 minutes at room temperature. Cells were filtered through a 35µm cell strainer, fixed and permeablized in Cytofix/Cytoperm buffer (BD Cytofix/Cytoperm™ Plus Fixation/Permeabilization Kit, BD Biosciences) during 20 min on ice. Next, BV-2 cells were washed and incubated on ice for 30 min in Perm/Wash buffer with fluorochrome-conjugated rat anti-mouse antibodies: iNOS-PE-Cy7 (clone CXNFT, eBioscience), Mac-2-PE (clone eBioM3/38, eBioscience) and IL1β-PE (clone 11n92, LifeSpan BioSciences). The following isotype controls were used: Rat IgG2aκ PE-Cy7, Rat IgG2aκ PE and Rat IgG2b PE (all from eBioscience). After washes, cells were resuspended in FACS buffer (PBS, 2% FCS, sodium azide), acquired in a FACS Aria II and analyzed with FACS Diva 6.1.3 software (BD Biosciences). Isotype-marker overlay graphs were created in FlowJo 10.0.8 Software.

1. **Supplementary Figures and Tables**

Supplementary Figure 1 shows the Mac-2, iNOS and IL1 beta reactivity in positive control BV-2 cells, indicating the capability of the antibodies to recognize the antigens.

## Supplementary Figures


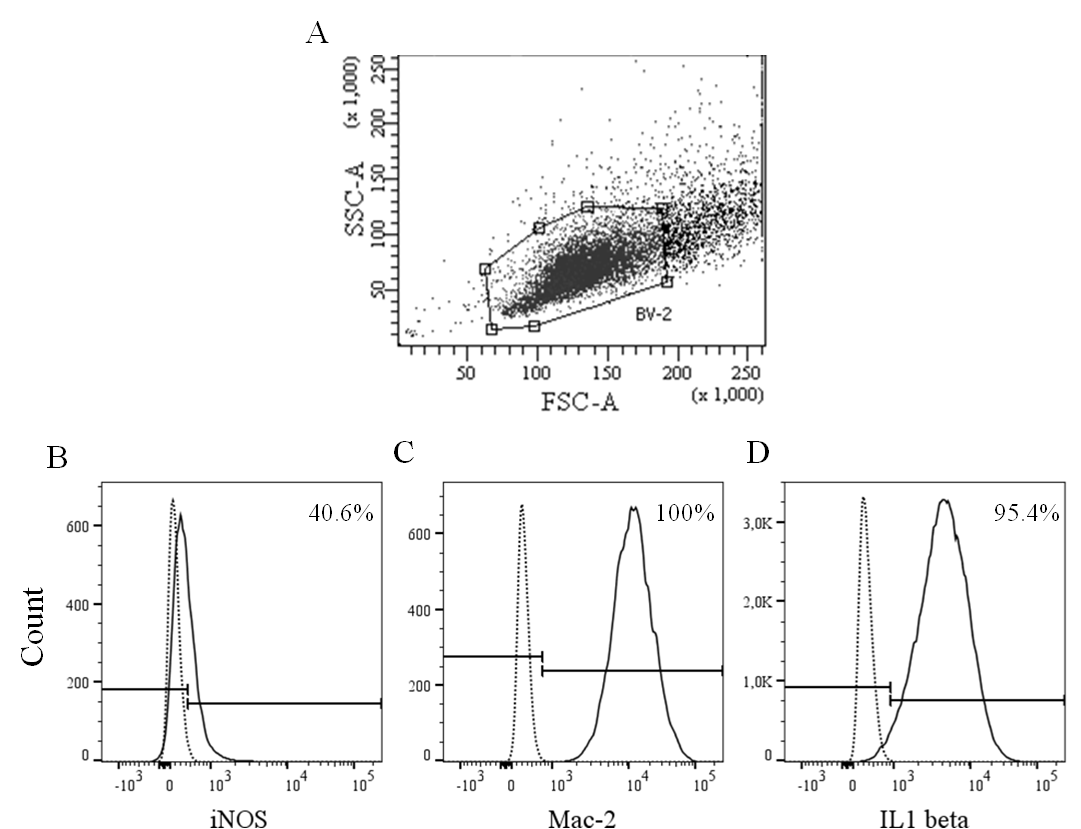


**Supplementary Figure 1. BV-2 cells are positive for iNOS, Mac-2 and IL1 beta (A-D).** BV-2 cells (A) were processed identical to the embryonic cortex cell suspension for flow cytometric staining. Using the same antibody concentration we find immune reactivity for iNOS (40.6%, B), Mac-2 (100%, C) and IL1 beta (95.4%, D) (full lines), which indicates that the antibody is capable to recognize the antigens. Gates for positive populations were drawn based on the isotype fluorescence intensity (dotted lines).
